# Activation of TRPV4 Induces Exocytosis and Ferroptosis in Human Melanoma Cells

**DOI:** 10.3390/ijms23084146

**Published:** 2022-04-08

**Authors:** Mei Li, Jiaojiao Zheng, Tian Wu, Yulin He, Jing Guo, Jiao Xu, Chuanzhou Gao, Shuxian Qu, Qianyi Zhang, Jiayu Zhao, Wei Cheng

**Affiliations:** Institute of Cancer Stem Cell, Dalian Medical University, Dalian 116044, China; limei791@dmu.edu.cn (M.L.); zheng810_phc@163.com (J.Z.); wutianhb1996@163.com (T.W.); hehylhyl@163.com (Y.H.); gjjing1020@163.com (J.G.); 17862666046@163.com (J.X.); gaocz@dmu.edu.cn (C.G.); qsx@dmu.edu.cn (S.Q.); zhangqy@dmu.edu.cn (Q.Z.); zhaojiayuofficial@163.com (J.Z.)

**Keywords:** TRPV4 ion channel, exocytosis, A375 cell, Ca^2+^ signaling, ferroptosis

## Abstract

TRPV4 (transient receptor potential vanilloid 4), a calcium permeable TRP ion channel, is known to play a key role in endocytosis. However, whether it contributes to exocytosis remains unclear. Here, we report that activation of TRPV4 induced massive exocytosis in both melanoma A375 cell and heterologous expression systems. We show here that, upon application of TRPV4-specific agonists, prominent vesicle priming from endoplasmic reticulum (ER) was observed, followed by morphological changes of mitochondrial crista may lead to cell ferroptosis. We further identified interactions between TRPV4 and folding/vesicle trafficking proteins, which were triggered by calcium entry through activated TRPV4. This interplay, in turn, enhanced TRPV4-mediated activation of folding and vesicle trafficking proteins to promote exocytosis. Our study revealed a signaling mechanism underlying stimulus-triggered exocytosis in melanoma and highlighted the role of cellular sensor TRPV4 ion channel in mediating ferroptosis.

## 1. Introduction

Exocytosis and its reversal process, endocytosis, are essential for maintaining cell health and proper physiological functions. Most cells use exocytosis to expel cytosolic contents, such as secretion proteins to the outside by fusion of vesicles with the plasma membrane [1]. During exocytosis, a vesicle budded from the endoplasmic reticulum (ER) or the Golgi apparatus is translocated along the cytoskeleton to the plasma membrane [2,3]. These vesicles either transport substances between the cell and its surroundings or provide a mechanism for remodeling the plasma membrane. While exocytosis is a precisely regulated process, for which calcium is known to play a critical role, the regulation mechanisms remain unclear.

Ferroptosis is a novel form of cell death characterized by accumulation of iron-dependent lipid reactive oxygen species (ROS) and disruption of plasma membrane unsaturated fatty acids [4,5,6]. Erastin induced ferroptosis involves in mitochondrial voltage-dependent anion channels [7], and studies indicate that PIEZO1 ion channel as well as chloride channels may mediate ferroptosis process [8,9,10]. Ferroptosis has been implicated in the pathogenesis of several diseases, including cancer, stroke, kidney degeneration, and so on [11,12]. However, the involvement of TRP ion channels in cancer cell death of ferroptosis is poorly understood.

TRPV4 is a member of the TRP ion channel superfamily [13,14]. Like most TRP channels, TRPV4 is highly permeable to calcium. TRPV4 can be activated by various physical or chemical stimuli; its activation leads to membrane depolarization and calcium influx. TRPV4 is widely distributed in both neuronal and non-neuronal tissues and organs, including brain, liver, intestine, olfactory epithelium, heart, lung, kidney, pancreas, bladder, testis, mammary gland, bone, and skeletal muscle [15]. Elevated TRPV4 expression has been noticed in certain tumors such as bladder, skin, stomach, and tuberous sclerosis [16,17,18,19]. Besides functioning in plasma membrane, TRPV4 also contributes to signaling in intracellular organelles. Distribution and trafficking of TRPV4 between intracellular organelles and plasma membrane are dynamically modulated by multiple factors [20,21,22,23,24]. Studies have shown that TRPV4 could bind to the ubiquitin ligase AIP4 to promote cell endocytosis, which in turn decreases TRPV4 distribution at the plasma membrane [25,26]; conversely, TRPV4 can also interact with PACSIN3 to increase plasma membrane distribution by inhibiting endocytosis [27,28]. However, very little is known about the involvement of TRPV4 in exocytosis and ferroptosis.

Here, we sought to investigate the role of TRPV4 in the process of exocytosis upon agonist stimulation in the A375 cell, which is a human melanoma cell line, as well as in several expression systems. We reasoned that if TRPV4 does actively modulate the exocytosis process in native cells, it might also do so in the heterologous expression system. We found that stimulation of native TRPV4 in A375 cells or transiently transfected TRPV4 in different cell lines indeed initiated prominent exocytosis. This cellular process requires TRPV4 as well as TRPV4-mediated calcium influx, lysosome associated protein, and several folding and vesicle trafficking proteins, all of which are key regulators of exocytosis. We also found that the ER primed vesicles upon agonist stimulation, whereas the subsequent mitochondrial crista reduction enabled calcium-dependent vesicular trafficking and induced cell ferroptosis. Our study identified a series of intracellular events following TRPV4 activation that facilitate exocytosis and revealed TRPV4 as a key functional link between exocytosis process and ferroptosis via calcium.

## 2. Results

### 2.1. Activation of TRPV4 Induced Prominent Exocytosis in Human Melanoma A375 Cells

**Figure 1:**
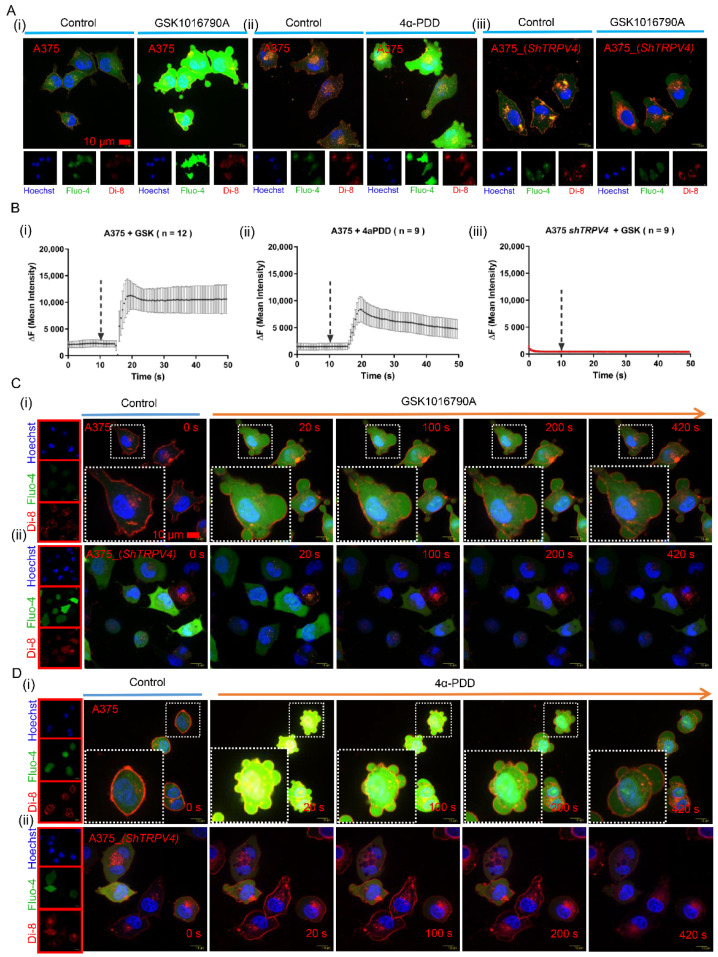
Activation of TRPV4 triggers exocytosis via agonist stimuli. (**A**) Representative scanning disk confocal images of agonists with (**i**) GSK1016790A (20 nM) or (**ii**) 4α-PDD (5 μM) stimuli to A375 cells and (**iii**) A375 cells with *TRPV4* knock-down. Fluorescence stained for nucleus (blue, hoechst 33342, 5 μg/mL), intracellular calcium (green, Fluo-4, AM, 1.25 μM), and plasma membrane (red, Di-8 ANEPPS, 10 μM). Scale bar, 10 μm. (**B**) Calcium imaging for agonists stimuli of TRPV4 by (**i**) GSK1016790A (20 nM), as well as (**ii**) 4α-PDD (5 μM) in human A375 melanoma cells and (**iii**) A375 cells with *TRPV4* knock-down. (**C**) Time-lapse of scanning disk confocal images of (**i**) A375 cells and (**ii**) controlled A375 cells with *TRPV4* knock-down at indicated time points after GSK1016790A (20 nM) administration. (**D**) Time-lapse of scanning disk confocal images of (**i**) A375 cells and (**ii**) A375 cells with *TRPV4* knock-down after 4α-PDD (5 μM) administration.

We previously found that TRPV4 ion channel is over-expressed in human melanoma A375 cells, and that activation of TRPV4 induced cell death [29]. Intriguingly, we observed prominent vesicle priming after administration of GSK1016790A, a TRPV4-specific activator, to A375 cells. However, human melanoma cells of A2058, as well as G361 with low expression of TRPV4, exhibited nearly imperceptible exocytosis. Based on these observations, we hypothesized that TRPV4 could have induced cell death by triggering excessive exocytosis in human melanoma. To test this hypothesis, A375 cells were incubated with Fluo-4 AM (to monitor intracellular calcium), Hoechst 33342 (to stain nucleus) as well as Di-8 ANEPPS (to label plasma membrane) for 1 hr with or without agonist stimulation of TRPV4. We observed that indeed GSK1016790A could induce prominent A375 cell exocytosis (Figure 1A(i)). Live cell imaging was confirmed rapid increase in intracellular calcium concentration upon TRPV4 activation (Figure 1B(i)). To confirm these findings, we used another TRPV4 activator, 4α-PDD (4α-phorbol 12, 13-didecanoate); similar agonist-induced exocytosis was observed in A375 cells (Figure 1A(ii)), accompanied by an increase in intracellular calcium concentration (Figure 1B(ii)). In contrast, agonist stimulation evoked only marginal responses in A375 cells whose expression of *TRPV4* was knocked down (Figure 1A(iii),B(iii)). These observations suggest that TRPV4 could be a key signal transducer in melanoma in response to cellular stimuli.

We further observed that the exocytosis process was time-dependent. Time-lapse imaging revealed that stimulation by either GSK1016790A (Figure 1C(i)) or 4α-PDD (Figure 1D(i)) led to a progressive increase of exocytic bubbles in A375 cells. No comparable exocytosis progression was observed in *TRPV4* knocked-down A375 cells (Figure 1C(ii),D(ii)). In addition, exocytosis reduced significantly when TRPV4 was inhibited by either ruthenium red (RR, a non-selective TRP channel blocker) or GSK2193874 (a specific channel blocker of TRPV4) before GSK1016790A was administrated to A375 cells (Supplementary Figure S1A,B). In summary, activation of endogenous TRPV4 in human melanoma A375 cells triggered prominent and progressive exocytosis, which likely led to eventual cell death.

### 2.2. Exocytosis Is Mediated by Agonist Stimulation in Exogenous TRPV4-Expressing Cells

**Figure 2:**
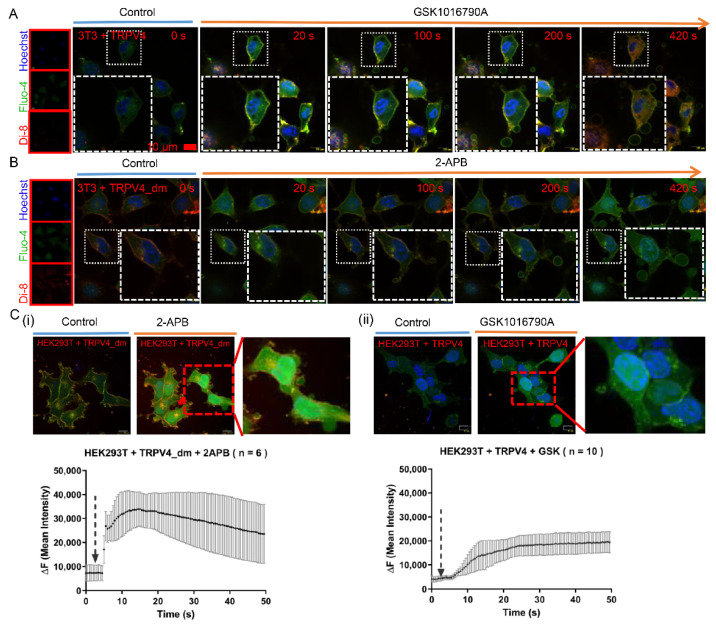

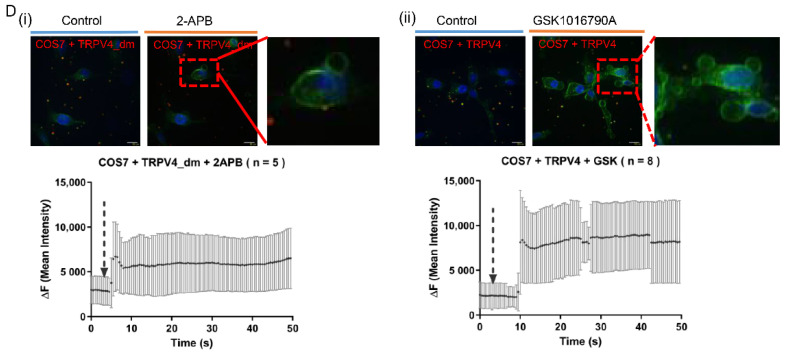
Functional TRPV4 induces cell exocytosis in heterogenous expression system. (**A**) Time-lapse of scanning disk confocal images of 3T3 cells transiently transfected with TRPV4 upon GSK1016790A (20 nM) exposure. (**B**) Time-lapse of scanning disk confocal images of 3T3 cells transiently transfected with TRPV4 carrying double mutations of N456H/W737R (TRPV4_dm) upon 2-APB (50 μM) application. (**C**) Representative scanning disk confocal images (upper row) and calcium imaging (lower row) of heterogenous TRPV4 mutation (**i**), as well as TRPV4 (**ii**) expressing in HEK293T cells (**C**) and COS7 cells (**D**) upon 2-APB (50 μM) (**i**) and GSK1016790A (20 nM) (**ii**) applications.

We next expressed TRPV4 in 3T3 cells to study the effects of agonist stimulation. We found that treatment with GSK1016790A was sufficient for vesicles production and did lead to exocytosis in exogenous TRPV4-expressing cells but not in vector-transfected controls (Figure 2A and Supplementary Figure S2A). 2-Aminoethoxydiphenyl Borate (2-APB) is a common activator for TRPV1, TRPV2, and TRPV3, whereas TRPV4, though sharing substantial sequence homologs with these temperature-sensitive thermoTRPs, is 2-APB insensitive. Introducing two point-mutations in TRPV4 (N426H in the cytoplasmic N-terminal and W737R in the cytoplasmic C-terminal TRP box) has been shown to be sufficient to introduce 2-APB sensitivity [30]. Remarkably, 3T3 cells transiently transfected with TRPV4 carrying the double mutations of N456H/W737R, termed TRPV4_dm, showed robust responses to 2-APB with exocytosis (Figure 2B) and intracellular calcium increases (Supplementary Figure S2C(i)). In contrast, 2-APB could not evoke exocytosis in wild-type TRPV4-expressing 3T3 cells (Supplementary Figure S2B) while GSK1016790A did (Supplementary Figure S2C(ii)).

To further confirm results obtained in 3T3 cells, we also exogenously expressed TRPV4 in both HEK293T and COS7 cells, for which agonist stimulation of TRPV4 could again induce cell exocytosis with increases in calcium influx (Figure 2C(i)–D(ii)). Therefore, activation of TRPV4 via agonist is responsible for the progressive exocytosis response in these as well as A375 melanoma cells.

### 2.3. TRPV4-Mediated Exocytosis in A375 Melanoma Cells Required Ca^2+^/CaM/CaMKII

**Figure 3:**
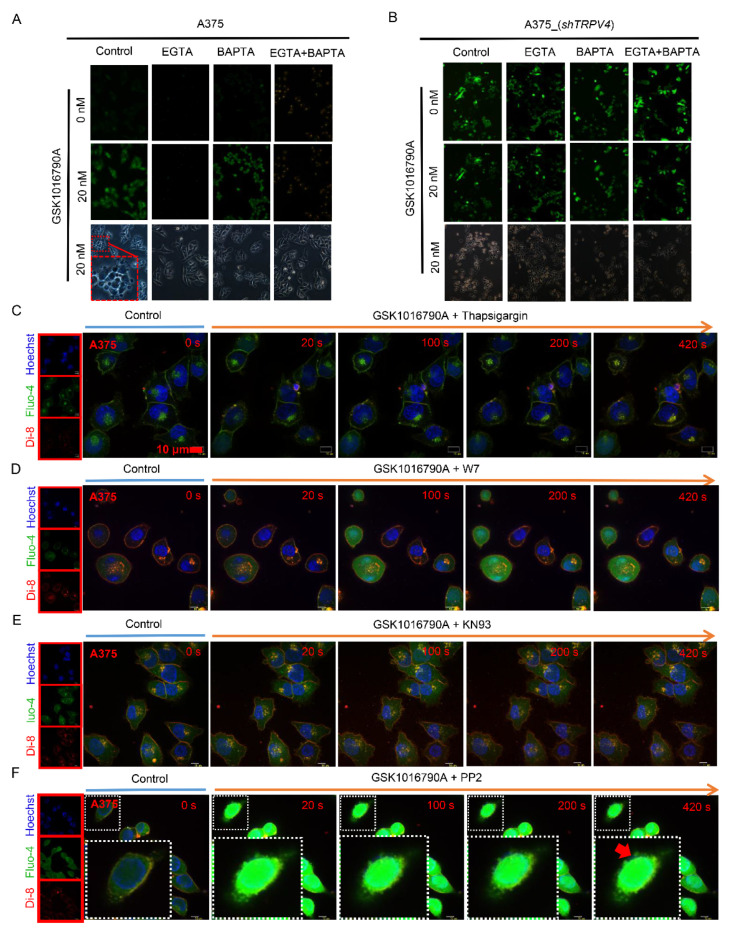
Blockade of Ca^2+^ signaling mediates exocytosis during activation of TRPV4 ion channel. (**A**) Cell images of A375 cells in GSK1016790A (20 nM) application with pretreatment by EGTA (0.5 mM) or BAPTA, AM (8 μM). (**B**) A375 cells in *shTRPV4* upon agonist stimuli with blockade of either extracellular or intracellular calcium. (**C**) Time-lapse of scanning disk confocal images of A375 cells upon GSK1016790A (20 nM) stimuli with pretreatment with thapsigargin (50 nM). (**D**) Time-lapse of scanning disk confocal images of GSK1016790A (20 nM) application to A375 cells with pretreatment with W7 (10 μM), a calmodulin antagonist. Scale bar, 10 μm. (**E**) Time-lapse of scanning disk confocal images of GSK1016790A (20 nM) application to A375 cells with pretreatment with KN93 (1 μM), a selective inhibitor of Ca^2+^/calmodulin-dependent kinase II. Scale bar, 10 μm. (**F**) Scanning disk confocal images for time-lapse in A375 cells upon GSK1016790A (20 nM) addition with pretreatment of PP2 (10 μM), an effective inhibitor of tyrosine kinases. Scale bar, 10 μm.

It is well-established that intracellular calcium signaling is necessary for the induction of endocytosis, and is also important for the initiation of vesicles for exocytosis [31,32,33]. Ionomycin was used to exclude calcium entry alone could not induce large membrane blebs (Supplementary Figure S3). Thus, we hypothesized that TRPV4-mediated calcium influx is likely involved in triggering exocytosis. We added EGTA (2 mM) to the extracellular solution and found that exocytosis triggered by GSK1016790A was substantially attenuated, supporting the idea that calcium entry from the extracellular environment is vital for the process. Addition of EGTA was also sufficient to block intracellular calcium increase (Figure 3A). Again, minimal exocytosis and negligible intracellular calcium increase were observed in *TRPV4* knock-down A375 cells upon GSK1016790A stimulation under this experimental condition (Figure 3B). In contrast, loading the cells with BAPTA (in the BAPTA AM form to allow uptake; cytosolic acetoxymethyl esters would cleave it to release the free acid form) did not affect intracellular content expulsion (Figure 3A). Furthermore, treatment with thapsigargin, an inhibitor of the sarco/endoplasmic reticulum calcium ATPase, showed similar reduction of cell exocytosis upon agonist stimuli (Figure 3C). These findings suggest that calcium entry from the extracellular side instead of release from the intracellular store is required for the TRPV4-mediated exocytosis events.

In order to identify the downstream events of calcium signaling during cell exocytosis, we pretreated A375 cells with W7 (a calmodulin antagonist), KN93 (a selective inhibitor of Ca^2+^/calmodulin–dependent kinase II) and PP2 (an effective inhibitor of tyrosine kinases). Exocytosis triggered by GSK1016790A was abrogated in the presence of W7 and KN93 (Figure 3D,E), but not of PP2 (Figure 3F). Thus, our data suggested that TRPV4 mediated exocytosis via Ca^2+^/CaM/CaMKII signaling pathway in A375 melanoma cells.

### 2.4. Accumulation of Lysosome-Associated Proteins Facilitated TRPV4-Mediated Exocytosis

Previous studies have revealed that exocytosis requires the lysosome as a key cargo store, and several vesicle trafficking proteins interact with lysosomes to regulate delivery of cargo throughout the cells [34,35,36,37,38]. Based on these findings, we decided to examine the potential involvement of specific lysosome-associated proteins in TRPV4-mediated exocytosis.

**Figure 4:**
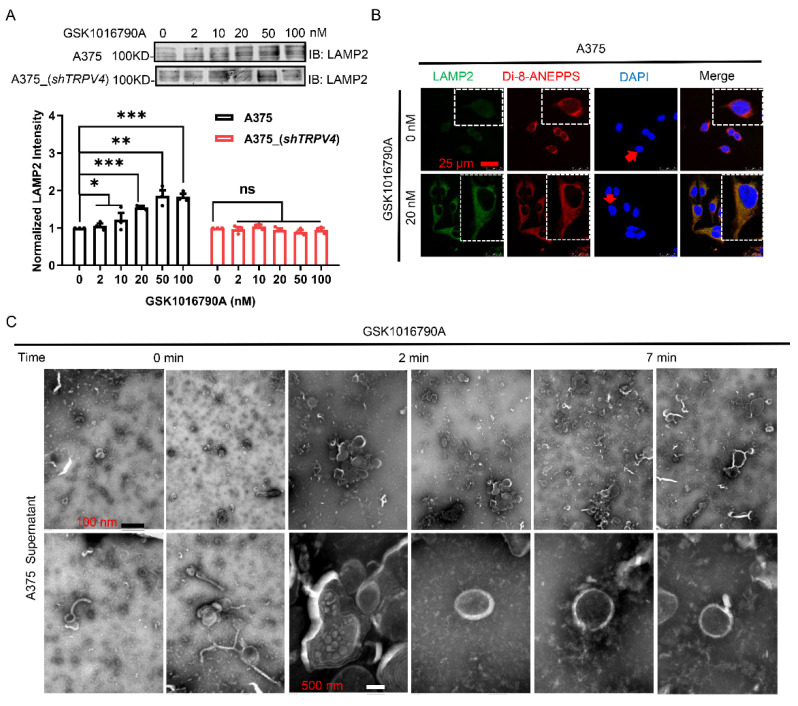
Lysosome-associated protein involves in TRPV4 mediated cell exocytosis. (**A**) Western blot of cell lysates probed for LAMP2 upon GSK1016790A (20 nM) in A375 cell as well as *shTRPV4* A375 cells. Target protein quantification was normalized to total protein. (**B**) Confocal microscopy images of A375 cells immunostained for LAMP2. Scale bar, 25 μm. (**C**) Transmission electron microscopic images of lysosomes in cell supernatant upon GSK1016790A (20 nM) application. Scale bar as indicated in figure. Data are presented as mean ± SD of at least three independent experiments, *t*-test, * *p* < 0.05, ** *p* < 0.01, *** *p* < 0.001, ns indicates no significance.

Given that lysosomal motility is known to be regulated by lysosome-associated membrane proteins (LAMP) during lysosome-phagosome fusion [39], a simple model would be that LAMPs may signal to lysosome by over-expression. However, we failed to detect elevation in the expression of LAMP1 during exocytosis induced by TRPV4 agonist stimulation (not shown). Though this does not completely rule out the possibility that LAMP1 may be involved in exocytosis in response to TRPV4 activation, it strongly suggests that LAMP1 probably regulates exocytosis indirectly. If lysosome motility is regulated by LAMPs, and LAMP1 is not involved, we reasoned that another protein must be involved in the TRPV4 pathway to regulate exocytosis, probably by acting upstream of exocytosis. Therefore, we considered that lysosome-associated membrane protein LAMP2 [40,41] may be a targeted protein in this case. Indeed, increased release of exocytosis after TRPV4 agonist stimulation was accompanied with up-regulation of LAMP2 in human melanoma A375 cells (Figure 4A,B). In contrast, after knocking down *TRPV4* expression, no increase in LAMP2 could be observed (Figure 4A). In transmission electron microscopy (TEM) images, elevated lysosomes (Figure 4C) were observed from GSK1016790A treated cells. Our results, thus, identified LAMP2 accumulation in lysosomes as a key event during TRPV4-mediating exocytosis in A375 cells.

### 2.5. Cellular Exocytosis Response Is Aided by Recruitment of Folding and Vesicle Trafficking Proteins

**Figure 5:**
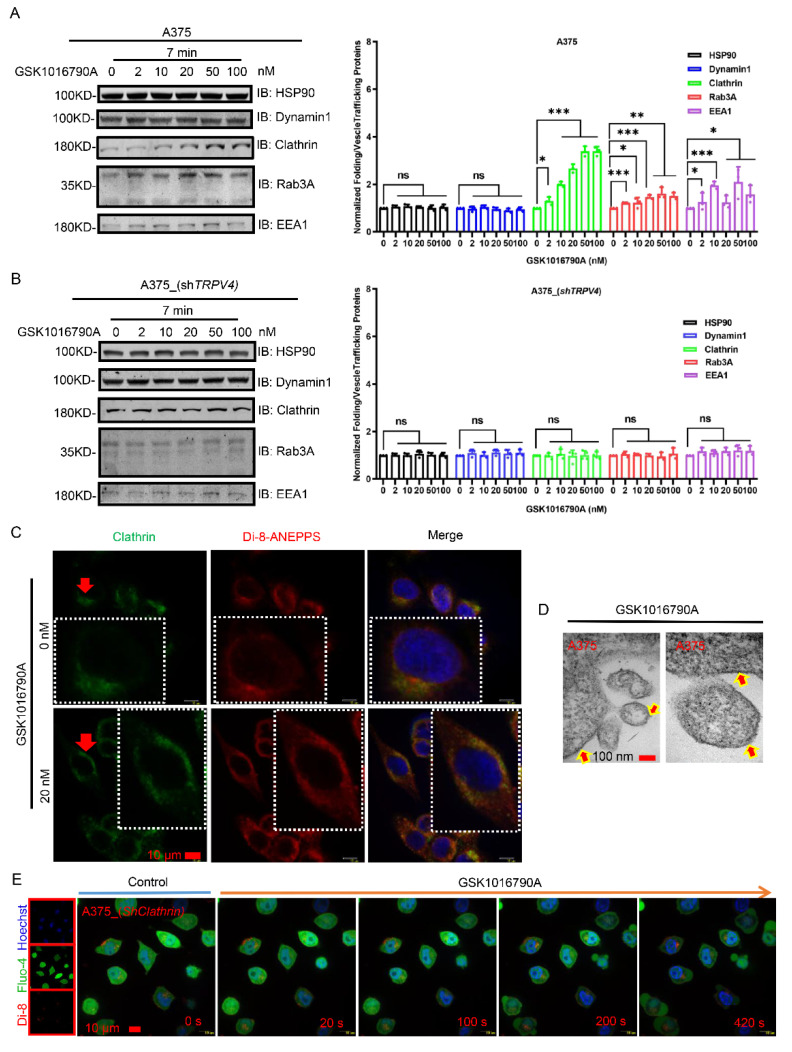
The folding & vesicle trafficking proteins aid in exocytosis. (**A**) Western blot of A375 cell (upon GSK1016790A by concentration gradient) lysates probed for the vesicle trafficking proteins of HSP90, dynamin1, clathrin (heavy chain), Rab3A, and EEA1, respectively. Target protein quantification was normalized to total protein. (**B**) Western blot of A375 cell with *shTRPV4* (upon GSK1016790A by concentration gradient) lysates probed for the vesicle trafficking proteins of HSP90, dynamin1, clathrin (heavy chain), Rab3A, and EEA1, respectively. Target protein quantification was normalized to total protein. (**C**) Confocal spanning disk microscopic images of A375 cells with or without GSK1016790A (20 nM) addition immunostained for clathrin (heavy chain). Scale bar, 10 μm. (**D**) Transmission electron microscopic images of A375 cells upon GSK1016790A (20 nM) application. The arrowhead points to clathrin structure. Scale bar, 100 nm. (**E**) Time-lapse of scanning disk confocal images of A375 cells with *clathrin* (heavy chain) knock-down with GSK1016790A (20 nM) administration. Data are presented as mean ± SD of at least three independent experiments, *t*-test, * *p* < 0.05, ** *p* < 0.01, *** *p* < 0.001, ns indicates no significance.

Multiple cellular proteins participating in folding and vesicle trafficking have been identified to be involved in exocytosis. We examined several key vesicular markers, including clathrin, dynamin1, EEA1, HSP90, and Rab3A. Indeed, when agonist treatment of TRPV4–expressing A375 cells caused exocytosis, there were increased expressions of Rab3A, EEA1, as well as clathrin, in a GSK1016790A concentration-dependent manner, whereas the level of HSP90 and dynamin1 remained unchanged (Figure 5A). Silencing of *TRPV4* in A375 cells clearly wiped out the change of trafficking proteins induced by agonist stimulation (Figure 5B). Activation of TRPV4 by GSK1016790A could induce an augment of clathrin into plasma membrane, which could be detected by both immunofluorescence and by TEM imaging (Figure 5C,D). To further confirm the involvement of clathrin, we knocked down the expression of *clathrin* in A375 cells. We observed that in this case, agonist stimulation evoked only marginal exocytosis (Figure 5E). Therefore, clathrin and other vesicle trafficking proteins are recruited during TRPV4 activation induced exocytosis.

### 2.6. Vimentin and SNX9 Are Up-Regulated in Cellular Exocytosis after Aagonist Stimulation of TRPV4

**Figure 6:**
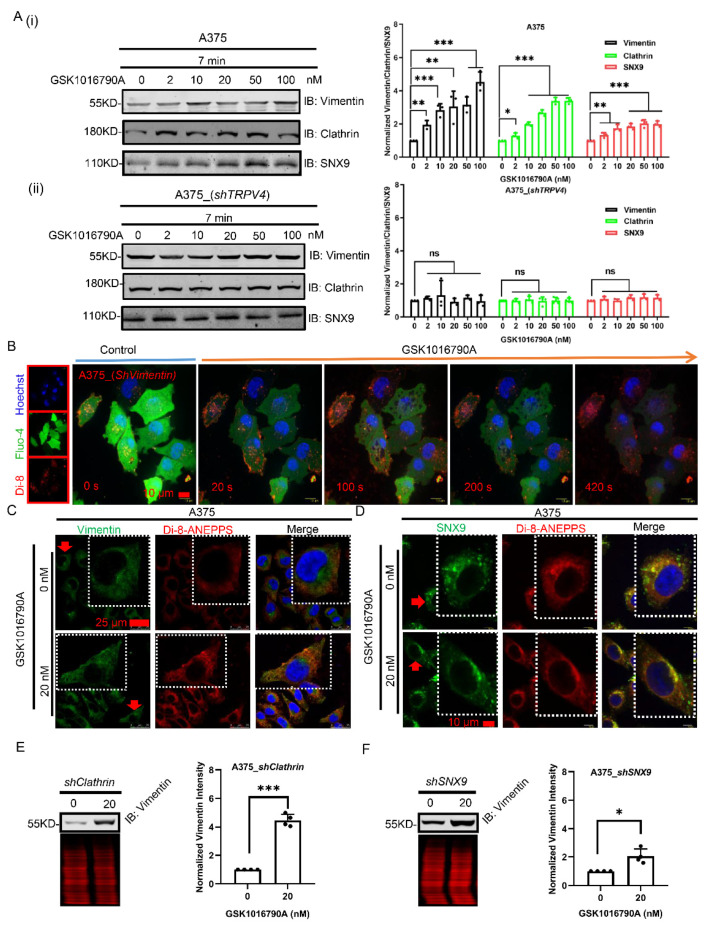
The interplay among vimentin/clathrin/SNX9 aids vesicles transduction of exocytosis. (**A**) Western blot of A375 and A375 with suppression of *TRPV4* (upon GSK1016790A by concentration gradient) cell lysates probed for vimentin, clathrin (heavy chain), and SNX9, respectively. Target protein quantification was normalized to total protein. (**B**) Time-lapse of scanning disk confocal images of A375 cells with *vimentin* knock-down with GSK1016790A (20 nM) administration. Scale bar, 10 μm. (**C**) Confocal microscopic images of A375 cells treated with or without GSK1016790A (20 nM) immunostained for vimentin. Scale bar, 25 μm. (**D**) Confocal spanning disk microscopic images of A375 cells treated with or without GSK1016790A (20 nM) immunostained for SNX9. Scale bar, 10 μm. Western blot of A375 with (**E**) *shclathrin* and (**F**) *shSNX9* (upon GSK1016790A (20 nM) stimuli) cell lysates probed for vimentin. Target protein quantification was normalized to total protein. Data are presented as mean ± SD of at least three independent experiments, *t*-test, * *p* < 0.05, ** *p* < 0.01, *** *p* < 0.001, ns indicates no significance.

Besides clathrin, vimentin was also found to be increased during agonist stimulation of TRPV4 (Figure 6A(i)), whereas its expression level remained unchanged in *TRPV4*-silenced A375 cells (Figure 6A(ii)). In addition, subdued exocytosis was observed after TRPV4 agonist addition when *vimentin* was knocked down from A375 cells (Figure 6B). Immunofluorescent tests further confirmed increased vimentin expression at cell membrane in response to exocytosis induced by GSK1016790A (Figure 6C). These data suggest that vimentin plays a role in cellular release of vesicles.

Similarly, we observed that SNX9 expression also increased during agonist stimulation of TRPV4 (Figure 6A(i)) in a TRPV4-dependent manner (Figure 6A(ii)). TRPV4 agonist clearly enhanced SNX9 trafficking to the plasma membrane (Figure 6D). Previous studies indicate that SNX9 resides in the cytosol where it influences the processing and trafficking of insulin receptors [42]. The enzyme aldolase binds to and inactivates SNX9. Phosphorylation of SNX9 releases aldolase and frees SNX9 to recruit and activate dynamin II, a neuronal phosphoprotein and a GTPase enzyme which mediates late stages of endocytosis in both neural and non-neural cells [43]. In our present study, SNX9 was found to be also involved in exocytosis in A375 cell, yielding a more complete picture of the SNX9-mediated signaling process.

So far, our data reveal that the expression levels for clathrin, vimentin, and SNX9 increased during TRPV4 activation induced exocytosis in human melanoma A375 cells. We sought to identify the relationship among vimentin, clathrin, and SNX9 during the exocytosis process. For this purpose, we individually silenced each of these proteins. We found that the expression level of vimentin was still prominently elevated in both *shclathrin* and *shSNX9* of A375 cells after GSK1016790A treatment (Figure 6E,F). In contrast, expression of SNX9 in *shclathrin*, as well as expression of clathrin in *shSNX9* A375 cells after GSK1016790A treatment, remained unchanged (Supplementary Figure S4A,B). In summary, whereas both clathrin and SNX9 could affect vimentin expression in GSK1016790-treated A375 cells, neither clathrin nor SNX9 seems to affect each other. Therefore, clathrin or SNX9 inhibition alone cannot substitute for vimentin blockade in the process of exocytosis.

### 2.7. TRPV4 Mediates Exocytosis Priming from ER and May Result in Cell Ferroptosis

**Figure 7:**
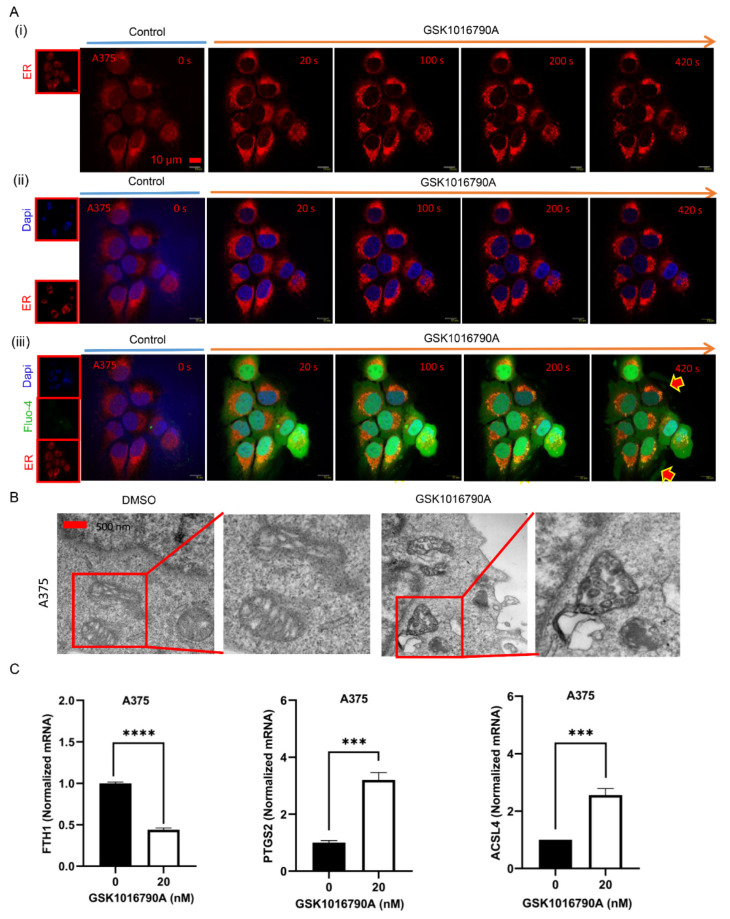
Agonism of TRPV4 induces vesicles priming from ER and subsequently ferroptosis of cells. (**A**) (**i**) & (**ii**) & (**iii**) Time-lapse of scanning disk confocal images of ER staining in A375 cells with GSK1016790A (20 nM) administration. Scale bar, 10 μm. (**B**) Transmission electron microscopic images of mitochondria in A375 cells incubated with GSK1016790A (20 nM) for 7 min. Scale bar, 500 nm. (**C**) Real-time PCR analysis of FTH1, PTGS2, and ACSL4 mRNA in A375 cells treated with GSK1016790A (20 nM). Data are presented as mean ± SD of at least three independent experiments, *t*-test, *** *p* < 0.001, **** *p* < 0.0001.

Vesicles often were priming from ER or Golgi apparatus. When treated with GSK1016790A, the ER in A375 cells harbored a high amount of priming vesicles (Figure 7A(i),A(ii)). Ca^2+^ signals have been accelerating significantly and widespread vacuolation were observed after ER activation (Figure 7A(iii)).

Interestingly, we observed a morphological change in mitochondria, with prominent reduction of mitochondrial crista found upon application of TRPV4 agonist (Figure 7B). Ferroptotic cells are characterized by changed mitochondrial morphology and cristae structure; thus, we examined several ferroptosis specific markers. Activation of TRPV4 significantly decreased iron metabolism-associated gene expression of FTH1 (ferritin heavy chain 1). In addition, ferroptosis drivers of ACSL4 (acyl-CoA synthetase long chain family member 4) and PTGS2 (prostaglandin-endoperoxide synthase 2) were found increased upon GSK1016790A addition (Figure 7C). Therefore, agonist stimulation of TRPV4 induces exocytosis and leads to cell ferroptosis.

## 3. Discussion

An earlier study identified that activation of TRPML3, another TRP channel localized to lysosomes, could spontaneously initiates lysosome exocytosis [44]. In the present study, we have demonstrated that agonist stimulation of TRPV4 strongly evokes exocytosis in A375 melanoma cells, which could be confirmed in multiple expression systems exogenously expressing TRPV4. Our results indicate that activation of TRPV4 dynamically regulates exocytosis. We have identified several molecules that play an important role in TRPV4 activation induced exocytosis. Notably, the process is also aided by skeleton protein of vimentin. Given that the mechanisms regulating exocytosis by activation of TRPV4 show striking conservation across different cells, our study raises the possibility that a similar phenomenon may also occur in other TRP distribution system.

### 3.1. TRPV4 Ion Channel and Calcium Signaling in Exocytosis

Our data suggest that activation of TRPV4 and the ensuing calcium influx via TRPV4 stimulate vesicles, which is facilitated by folding and vesicle trafficking proteins to the plasma membrane, leading to induction of exocytosis and ferroptosis. This finding uncovers a previously unrecognized function for TRPV4 ion channel.

Several potassium ion channels have been implicated in melanoma [45]. Ferrera and coworkers reported that potassium ion channels of BK and KCa3.1 were overexpressed in human melanoma cells, and activation of these ion channels were mediated by calcium influx through TRPM2 oxidation [46]. In our present study, exogenous expression of TRPV4 in HEK293T, 3T3, and COS7 cells (which are lack of BK and KCa 3.1 channel expression) demonstrated that activation of TRPV4 could directly induce prominent exocytosis, consistent with the hypothesis that activation of TRPV4 is the key in initiating cell exocytosis. In further support of this conclusion, we found drastically decreased exocytosis in A375 cells when *TRPV4* was knocked down. Moreover, the elevation of intracellular calcium, induced by either ionomycin (a Ca^2+^ ionophore), or Ca^2+^ release from endoplasmic reticulum (ER) have been excluded involving in cell exocytosis. Thus, the role of TRPV4 here appeared to be mediating calcium entry and generating a specific Ca^2+^ microdomain with subsequent calcium signaling transduction to facilitate cell exocytosis.

Calcium is well known as a key second messenger in a wide range of physiological processes [47,48,49]. In the present study, we identified that Ca^2+^/CaM/CaMKII constitute the downstream players of TRPV4 in participating in regulating exocytosis. Indeed, TRPV4 has been reported to regulate CaM and CaMKII in HeLa cells transfected with TRPV4, cultured primary mouse neurons, and in human mesenchymal stem cells [50,51,52]. Whether this calcium signaling involves in ferroptosis needs further elucidation.

### 3.2. The Interplay among TRPV4, Folding/Trafficking Proteins, and Skeleton Proteins

Here, we showed redistribution of several proteins as TRPV4-dependent cellular responses, providing a cellular correlate for agonist-induced exocytosis. Interestingly, one of the cellular responses is redistribution of TRPV4 itself from intracellular locations to the plasma membrane to potentiate calcium influx. Meanwhile, the increased vimentin helps vesicles trafficking to the plasma membrane. Clathrin and SNX9 aggregate to the plasma membrane to facilitate cell exocytosis. When TRPV4 expression was suppressed, redistribution of these proteins was also largely abolished. These findings demonstrated that TRPV4 acts upstream of folding and vesicle trafficking proteins, yet promoted exocytosis also leads trafficking of TRPV4 to plasma membrane. This positive feedback process, if unchecked, would lead to ferroptosis and tissue necrosis at least in human melanoma cells.

**Figure 8:**
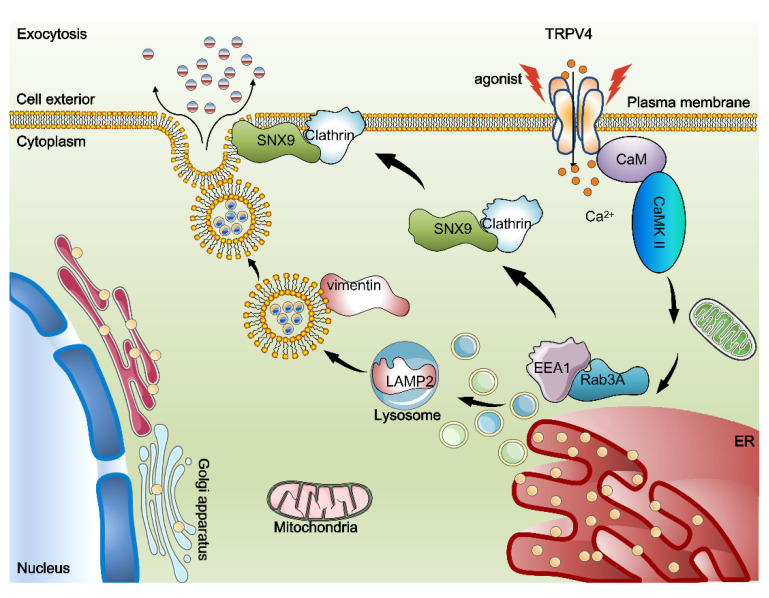
Proposed model of exocytosis by activation of TRPV4 upon agonist stimuli.

Although vimentin and clathrin, as well as SNX9, are all increased after TRPV4 activation, and silencing any one of them could suppress exocytosis. Vimentin was observed to aggregate after silencing either clathrin or SNX9, whereas no significant redistributions of either SNX9 or clathrin has been observed in response to suppression of the other two proteins (Supplementary Figure S4A–D). These findings indicate that vimentin may act upstream of clathrin and SNX9. Though both clathrin and SNX9 are considered as key factors in exocytosis; vimentin plays a special role in transferring vesicles, as well as in signaling to other proteins to coordinate the intracellular response (Figure 8).

### 3.3. TRPV4, Ferroptosis and Cellular Signalling

Ferroptosis is a unique modality of regulated necrotic cell death. It is driven by activation of the mitogen-activated protein kinase (MAPK) pathway in cancer cells [7]. Studies have been reported that activation of TRPV4 by agonist caused phosphorylation of ERK1/2, JNK1/2, and p38 MAPK [53,54,55], and repressed oxidative metabolism in adipocytes [54]. In our present study, TRPV4 agonism induced ferroptosis in melanoma A375 cell may involve in MAPK signaling. The hallmark of ferroptosis is unrestrained lipid peroxidetion. ACSL4 behaves as an important driver of ferroptosis through biosynthesis, remodeling of phosphatidylethanolamine, and activating lipid peroxidation. A study has reported that ACSL4 is required for ferroptotic cancer cell death via mediating production of 5-HETE (5-hydroxyeicosatetraenoic acid) [56]. In the present study, ACSL4 exhibited increases upon agonism of TRPV4 for ferroptosis execution. Prostaglandin-endoperoxide synthase (PTGS) is the key enzyme in prostaglandin biosynthesis. There are two isozymes of PTGS: PTGS1 and PTGS2. Upregulation of PTGS2 indicates ferroptosis onset [57]. Indeed, our data confirmed that increased PTGS upon TRPV4 agonism. Together, our study indicated that ferroptosis is aided by TRPV4 ion channel signaling, while future studies are needed to elucidate the detailed molecular mechanisms.

In summary, our study uncovered a role of TRPV4 in mediating exocytosis and ferroptosis, and revealed a signaling pathway that integrating key players previously known to participate in exocytosis. Similar to TRPV4, many other TRP ion channels are calcium permeable and widely expressed in neurons as well as non-excitable tissues [58,59,60,61]. Whether these channels regulate exocytosis, as well as ferroptosis, in other cell types remains to be examined. Nonetheless, the present study offers yet another example in which a cellular sensor TRP ion channel actively contributes to a cellular defense mechanism against noxious stimuli.

## 4. Materials and Methods

### 4.1. Antibodies and Chemical Regents

All antibodies in the present study were obtained from Pierce Biotechnology or CST (Cell Signaling Technology, Danvers, MA, USA). All chemical agents were purchased from Sigma (Merck, St. Louis, MO, USA).

### 4.2. Cell Culture and Transfection

Human melanoma cell line of A375 (ATCC^®^ CRL-1619), human embryonic kidney cell line of HEK293T (ATCC^®^ CRL-11268), Cercopithecus aethiops kidney cell line of COS-7 (ATCC^®^ CRL-1651), and mouse embryo cell line of NIH/3T3 (ATCC^®^ CRL-1658) were all purchased from American Type Culture Collection (ATCC, Manassas, VA, USA). These four cell lines were cultured in DMEM (ATCC) supplemented with 10% fetal bovine serum (FBS, Gibco, Grand Island, NY, USA). All cells were grown in a humidified incubator at 37 °C in 5% CO_2_ atmosphere. Cells were transiently transfected with plasmids using Lipofectamine 3000 (Invitrogen, Carlsbad, CA, USA) following manufacturer’s instruction. After 1–2 days, expressed cells were chosen to perform calcium imaging or other functional experiments.

### 4.3. Western Blot

Cells were rinsed twice with phosphate-buffered saline (PBS) and then lysed on ice in 200 μL cold cell lysis buffer for Western test containing 20 mM Tris-HCl (pH 7.5), 150 mM NaCl, 1% Triton X-100, 2.5 mM sodium pyrophosphate, 1 mM β-glycerophosphate, 1 mM EDTA, 0.5 μg/mL leupeptin, and 1 mM phenylmethylsulfonyl fluoride (PMSF). The lysate was centrifuged at 14,000× *g* at 4 °C for 5 min. The supernatant was stored at −80 °C as whole-cell protein extracts. Protein concentrations were determined by BCA Protein Assay Kit. Proteins were separated by 10% SDS-PAGE gel and then transferred to nitrocellulose filter (NC) membrane. The NC membrane was incubated with Total Protein Stain (REVERT total protein stain, Li-Cor Biosciences, Lincoln, NE, USA, 926-11015) for 1 min and detected by Odyssey at 700 nm wavelength. The NC membrane was blocked with 5% non-fat milk in TBST (10 mM Tris-HCl, pH 8.0, 150 mM NaCl and 0.05% Tween-20) for 1 h at room temperature. The NC membrane was then incubated with primary antibody (1:500 dilution) in TBST at 4 °C overnight. After incubation with IRDye 800 CW Goat Anti-Rabbit IgG (H+L) (1:10000 dilution) in 5% non-fat milk in TBST for 40 min at room temperature, blots were detected using Odyssey. Target protein quantification was normalized to total protein. All Western blots were repeated at least three times for each experiment to confirm reproducibility.

### 4.4. Transmission Electron Microscopy

A375 cells (treatment with GSK1016790A for 7 min) were digested with trypsin and centrifuged at 3000× *g* for 5 min, immediately prefixed with a fixation solution of 2.5% glutaraldehyde at room temperature (25 °C) placed at 4 °C for more than 2 h. The cell pellet was aspirated with glutaraldehyde and rinsed with 0.1 M fresh phosphate buffer (PBS) for 15 min; this process was repeated three times. The vitrified sample was transferred to a fume hood and fixed with 1% osmium tetroxide for 2 h. The fixative was removed and the cell pellet rinsed three times with PBS for 15 min each, followed by dehydration with 50%, 70%, 80%, 90%, and 100% ethanol 15 min washing, respectively. The sample was infiltrated with 1,2-epoxypropane for 10 min and epoxy resin (Sigma) overnight in 4 °C refrigerator. The mixture of propylene oxide and epoxy resin was poured out and the pellet was refilled with epoxy resin using a glass rod and incubated at 4 °C for 4 h. The embedded sample was dissected with an EM UC6 ultramicrotome (Leica Microsystems) using a glass knife. Sections were placed on formvar-coated copper-rhodium slot grids (Electron Microscopy, Beijing, China) and stained with 2% uranyl acetate and lead citrate, and imaged with a JEM-2000EX transmission electron microscope (300 KeV) equipped with a CCD camera (JEOL, Tokyo, Japan).

Negative staining was used for cell supernatant analysis. The cell supernatant (7 min after GSK1016790A application to A375 cells) was collected in a centrifuged tube and ultra-centrifuged at 100,000× *g* for 1.5 h at room temperature. The supernatant was discarded and resuspended with 10–20 μL PBS. The sample was added to a 200 mesh copper EM grids with a carbon-coated formvar film (15 min). The copper mesh was then stained with 3% phosphotungstic acid for 5 min. The excess liquid was removed after the sample was dried in a 1.5 mL tube at 4 °C The sample was observed via a JEM-2000EX transmission electron microscope.

### 4.5. Intracellular Calcium Measurements

Calcium imaging was performed as previous described [29] to determine the intracellular calcium dynamics using Fluo-4 Direct Calcium Assay Kit (Invitrogen) according to manufacturer’s protocol. The fluorescence was measured at an excitation wavelength of 488 nm and emission wavelength of 525 nm. Data were analyzed with MetaMorph (NX 2.0, Molecular Devices, San Jose, CA, USA) software.

### 4.6. Post Transcriptional Gene Silencing

Gene silencing was used to knock down targeted proteins of interest (Table 1). The Dhamacon GIPZ lentiviral *shRNA* was purchased from GE which has been bioinformatically verified to match NCBI sequence data. Plasmid was purified using the Midi Kit (Qiagen, Duesseldorf, Germany). The sequences as the sense strand are shown in Table 1. Transfection of *shRNA* was performed according to manufacturer’s protocol. The empty vector GIPZ Lentiviral *shRNA* was used as a negative control. Silencing efficiency was determined by immunoblot analysis.

### 4.7. Imunostaining and Imaging

Cells on glass coverslips were fixed with 4% Paraformaldehyde solution for 10 min at 36 h post-transfection. They were permeabilized in 0.1% Triton for 10 min and blocked in 2% BSA for 1 h. Primary antibodies were used in a 1:1000 dilution against LAMP2, clathrin, vimentin, and SNX9, respectively, and incubated overnight at 4 °C. Secondary antibodies of Fluorescein (FITC)-labeled goat anti-Rabbit IgG (H+L) (A0562, Beyotime, Shanghai, China), and Fluorescein (FITC) goat anti-mouse IgG (A0568, Beyotime, Shanghai, China) were used at 1:500 dilution. All antibodies were diluted in blocking buffer and incubated for 1 h at room temperature. Nuclear was stained with dapi (D1306, Invitrogen, Carlsbad, CA, USA) for 5 min. Di-8 ANEPPS (D3167, Invitrogen), a red fluorescent dye that bound specifically to the outer leaflet of the plasma membrane, was used as a marker for the plasma membrane. Cells were observed with a Leica TCS-SP5 confocal microscope with a 63× 1.40 immersion oil objective, or with a spinning disk confocal microscope with a 60× 1.40 immersion oil objective.

### 4.8. Time-Lapse Microscopy

Time-lapse images were acquired using a confocal spinning disk (Yokogawa CSU-X, Tokyo, Japan) mounted on an inverted microscope equipped with an autofocus system (Ti-ECLIPSE; Nikon, Tokyo, Japan). Light sources used for fluorescence imaging included an argon ion laser (300 mW; Melles Griot), three diode-pumped solid-state lasers including 405 nm (100 mW; Andor, Belfast, Northern Ireland), 561 nm (50 mW; Andor), and 640 nm (100 mW; Andor). Images were captured with a high-sensitivity iXon Ultra EM-CCD camera (DU-897U; Andor) operated with the Andor iQ3 software (Andor). Cells were seeded on glass coverslips and recorded before and after TRPV4 agonist (20 nM GSK1016790A or 5 μM 4α-PDD) application. The confocal images of labeled cells were collected using a 60× oil objective. Nuclear was stained with hoechst 33342 (H1399, Invitrogen) for 10 min. Di-8 ANEPPS (D3167, Invitrogen) was applied for 20 min as a marker for plasma membrane. Fluo-4, AM was used to measure intracellular Ca^2+^, for which cells were incubated in the dye solution at room temperature for 1 h.

### 4.9. Real-Time PCR Validation

Total RNA was extracted from cultures of human melanoma A375 cells using RNAiso plus Kit (Takara, Tokyo, Japan). Then, the reverse transcription was performed with PrimeScript RT reagent Kit with gDNA Eraser (Takara). Primer and probe sequences were designed using Primer-Blast of NCBI. To determine ferroptosis in A375 cells upon agonist stimuli, real-time PCR was performed with the CFX Connect Real-Time PCR system (Bio-rad, Berkeley, CA, USA) using SYBRGreen Kit (Takara). Primer sequences are given in Table 2.

### 4.10. Statistical Analysis

Statistical results are expressed as mean ± standard deviation (SD). Statistical significance was evaluated by one-tailed Student’s *t*-test. All statistical tests were performed via GraphPad Prism 5. Significance was set at * *p* < 0.05; ** *p* < 0.01; or *** *p <* 0.001; ****p < 0.0001.

## Figures and Tables

**Table 1:** Sense Strand of Sequences for Posttranscriptional Gene Silencing.

Gene	Sense Strand	Entrez Gene ID
*TRPV4*	5′-ACCAAGTTTGTTACCAAGA-3′	59341
*Clathrin*	5′-GTGCTCTATTTATATAGAA-3′	1213
*Vimentin*	5′-ACACTCAGTGCAGCAATAT-3′	7431
*SNX9*	5′-CTAACACCTACTAACACTA-3′	51429

**Table 2:** Primer Sequences of Ferroptosis-Specific Primers Used in Real-Time PCRs.

Gene	Forward Primer	Reverse Primer	NM-Number
FTH1	AGAACTACCACCAGGACTCAGA	CAAAGCCCACATCATCGCGG	NM-002032.3
ACSL4	TTTTGCGAGCTTTCCGAGTG	AGCCGACAATAAAGTACGCC	NM-022977.3
PTGS2	CTGATGATTGCCCGACTCCC	CGCAGTTTACGCTGTCTAGC	NM-000963.4
GPX4	TGGACGAGGGGAGGAGC	TCGATGTCCTTGGCGGAAAA	NM-002085.5
NOX1	TAAAGGCTCACAGACCCTGC	GGCCAATGTTGACCCAAGGA	NM-007052.5
β-actin	TGGCATCCACGAAACTACCTT	TCGTCATACTCCTGCTTGCTG	NM-001101.3

## Data Availability

Original data are available from the authors on request.

## Supplementary Materials

The following supporting information can be downloaded at https://www.mdpi.com/article/10.3390/ijms23084146/s1.
