# Supplementary material for: Activation of TRPV4 Induces Exocytosis and Ferroptosis in Human Melanoma Cells
Source: Int J Mol Sci. 2022 Apr 8;23(8):4146. doi: 10.3390/ijms23084146 (PMC9030060; doi:10.3390/ijms23084146)
Supplement: Supplementary file 1 [file ijms-23-04146-s001.zip › ijms-1622778-supplementary.pdf]

International Journal of *Molecular Sciences*

MDPI

Supplementary data

Activation of TRPV4 Induces Exocytosis and Ferroptosis in Human Melanoma Cells

Mei Li †, Jiaojiao Zheng †, Tian Wu †, Yulin He †, Jing Guo, Jiao Xu, Chuanzhou Gao, Shuxian Qu, Qianyi Zhang, Jiayu Zhao and Wei Cheng *

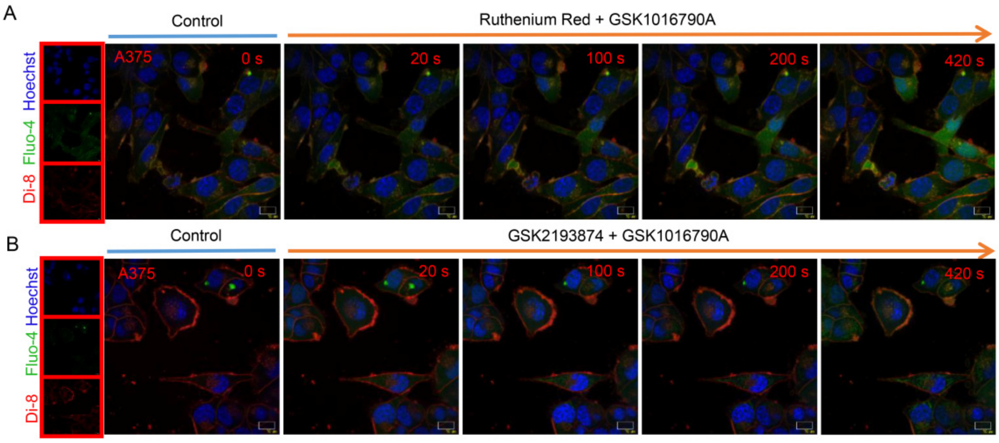

Supplementary Figure S1. Cell exocytosis in A375 cells targets TRPV4 pathway. (**A**) Scanning disk confocal images for time-lapse in A375 cells upon GSK1016790A (20 nM) addition with pretreatment of ruthenium red (10 μM), a common inhibitor of TRPV ion channels. Scale bar, 10 μm. (**B**) Scanning disk confocal images for time-lapse in A375 cells upon GSK1016790A (20 nM) addition with pretreatment of GSK2193874 (8 nM), an antagonist of TRPV4 ion channel. Scale bar, 10 μm.

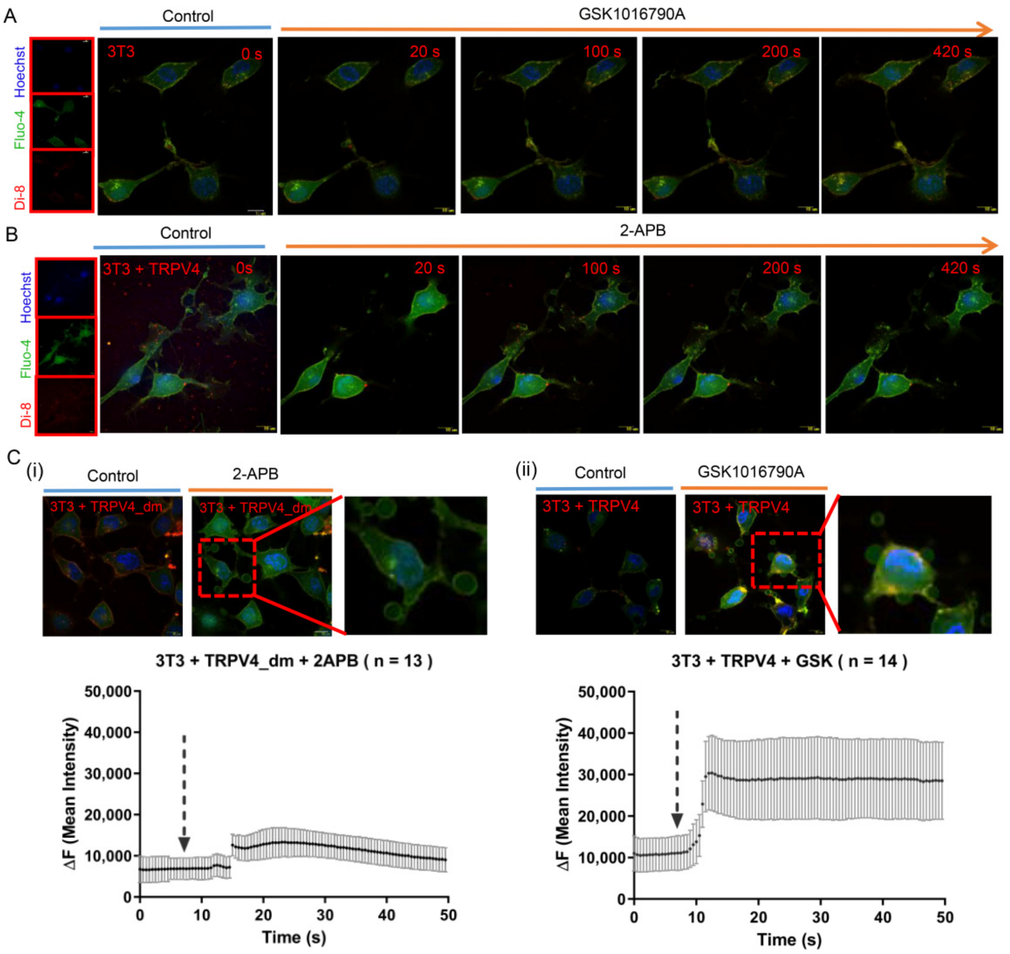

Supplementary Figure S2. Activation of TRPV4 induces cell exocytosis in exogenous expressing system. (**A**) Time-lapse of scanning disk confocal images of 3T3 cells transiently transfected with vector upon GSK1016790A (20 nM) application. (**B**) Time-lapse of scanning disk confocal images of 3T3 cells transfected with TRPV4 upon 2-APB (50 μM) application. (**C**) Representative scanning disk confocal images (upper row) and calcium imaging (lower row) of heterogenous TRPV4 mutation (**i**), as well as TRPV4 (**ii**) expressing in 3T3 cells upon 2-APB (50 μM) (**i**) and GSK1016790A (20 nM) (**ii**) application.

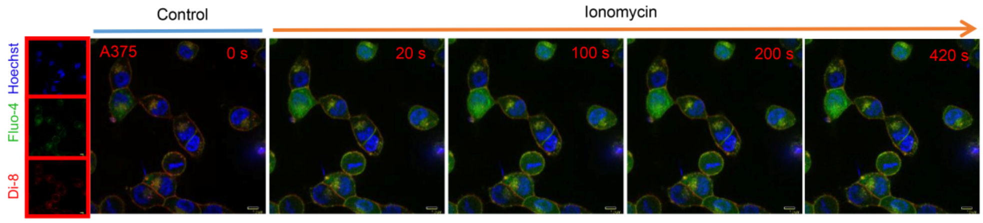

Supplementary Figure S3. Calcium entry alone does not induce cell exocytosis in A375 cells. Time-lapse of scanning disk confocal images for A375 cells upon application of ionomycin (1 μM), an effective Ca^{2+} ionophore. Scale bar, 10 μm.

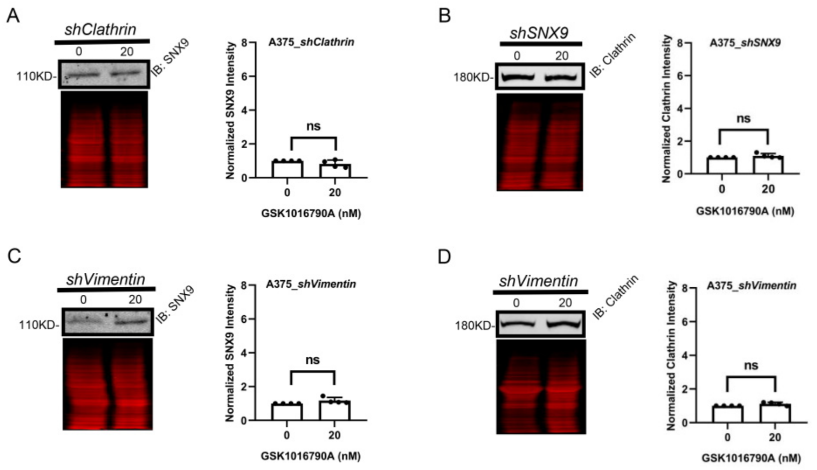

Supplementary Figure S4. The interplay among folding and vesicle trafficking proteins aids vesicles transduction of exocytosis. Western blot probed for SNX9 in A375 cells with *shclathrin* (**A**) and clathrin with *shSNX9* (**B**) treated with GSK1016790A (20 nM). Target protein quantification was normalized to total protein. Western blot probed for SNX9 (**C**) or clathrin (**D**) in A375 cells with *shvimentin* exposed to GSK1016790A (20 nM). Target protein quantification was normalized to total protein. Data are presented as mean ± SD of at least three independent experiments, *t*-test, ns indicates no significance.
